# Relationship between digital information and thermodynamic stability in bacterial genomes

**DOI:** 10.1186/s13637-016-0037-x

**Published:** 2016-02-02

**Authors:** Dawit Nigatu, Werner Henkel, Patrick Sobetzko, Georgi Muskhelishvili

**Affiliations:** 1 Transmission Systems Group, School of Engineering and Science, Jacobs University Bremen, Campus Ring 1, Bremen, 28759 Germany; 2 Philipps-Universität Marburg, LOEWE-Zentrum für Synthetische Mikrobiologie, Hans-Meerwein-Straße, Mehrzweckgebäude, Marburg, 35043 Germany; 3 Microbiologie, Adaptation, Pathogénie, UMR5240 CNRS-UCBL-INSA-BayerCropScience, Lyon, France; 4 Jacobs University Bremen, Campus Ring 1, Bremen, 28759 Germany

**Keywords:** Sequence analysis, Thermodynamic stability, Shannon entropy, Gibbs entropy

## Abstract

Ever since the introduction of the Watson-Crick model, numerous efforts have been made to fully characterize the digital information content of the DNA. However, it became increasingly evident that variations of DNA configuration also provide an “analog” type of information related to the physicochemical properties of the DNA, such as thermodynamic stability and supercoiling. Hence, the parallel investigation of the digital information contained in the base sequence with associated analog parameters is very important for understanding the coding capacity of the DNA. In this paper, we represented analog information by its thermodynamic stability and compare it with digital information using Shannon and Gibbs entropy measures on the complete genome sequences of several bacteria, including *Escherichia coli* (*E. coli*), *Bacillus subtilis* (*B. subtilis*), *Streptomyces coelicolor* (*S. coelicolor*), and *Salmonella typhimurium* (*S. typhimurium*). Furthermore, the link to the broader classes of functional gene groups (anabolic and catabolic) is examined. Obtained results demonstrate the couplings between thermodynamic stability and digital sequence organization in the bacterial genomes. In addition, our data suggest a determinative role of the genome-wide distribution of DNA thermodynamic stability in the spatial organization of functional gene groups.

## Introduction

The double-helical DNA polymer is the carrier of the genetic information required for the reproduction of any organism. This information is inscribed by the sequence of the four bases adenine (A), thymine (T), cytosine (C), and guanine (G) paired in a complementary fashion (A with T and G with C). The unique succession of the base pairs (letters) in a gene dictating the production of RNA molecules and proteins provides for digital type of information. The digital nature of the genetic code can be also seen in the correspondence of the “on-or-off” type digital logic with the feature that the genes can be expressed or not [1]. However, there is another type of information, dubbed “analog code”, that coexists with the digital code and is related to physicochemical properties of the DNA [1, 2]. This three-dimensional information emerges as a result of dynamic structural and topological variations of the chromosomal DNA and is involved in facilitating and regulating the gene expression, chromosome compaction, and replication [3–5]. The analog nature of this information is obvious because it is the additive interactions of successive base steps rather than individual base pairs which determine the physicochemical properties of the polymer. These properties, including DNA thermodynamic stability and supercoiling, are by definition the continuous properties that play a central role in determining the strength of gene expression [3].

The two types of information are intrinsically coupled by the primary DNA sequence. The physicochemical properties characterizing the analog information are largely sequence dependent. Preferred direction for bending (anisotropy), stiffness, thermodynamic stability, and supercoiling are among the properties that are essentially dependent on the DNA sequence organization [3, 6, 7]. Previous studies provided compelling arguments concerning the peculiar relationship of interdependence between the two types of DNA information [1–4, 8, 9].

The average information content of the genome can be measured using Shannon entropy [10]. So far, researchers have extensively applied this information-theoretic measure for studying a wide variety of topics in molecular biology and bioinformatics, including DNA pattern recognition, gene prediction, sequence alignment, and comparative genomics [11–18]. Shannon entropy can be applied to bacterial organisms for analyses of the underlying digital coding device. However, because of the existence of an equally important analog code, we believe that solely looking at the base or codon composition in DNA sequences will miss the complete description of the underlying coding structure. For this, it is vital to look jointly into both the digital and analog information types encoded in the nucleotide sequence.

It is asserted that the relative stability of the DNA duplex structure relies on the identity of successive base steps [19, 20]. Stacking between adjacent base pairs and pairing between complementary bases determine the thermodynamic stability of the DNA [21, 22]. Since the stability of the DNA appears as a decisive factor in most of the biological processes, and due to the availability of thermodynamic parameters to describe DNA stability, such as Santalucia’s unified nearest-neighbor (NN) thermodynamic stability parameters (free energies) of Watson-Crick base pairs in 1 M NaCl [23], we assume relative thermodynamic stability as a measure of analog information.

We already made a first attempt in direction of integrating the digital and analog codes [24]. In this study, we base our analysis and observations on four selected bacterial genomes, namely *Escherichia coli K12 MG1655* (accession NC_000913), *Bacillus subtilis subsp. subtilis str. 168* (accession NC_000964), *Salmonella enterica subsp. enterica serovar Typhimurium DT104* (accession NC_022569), and *Streptomyces coelicolor A3(2)* (accession NC_003888). The general goal is to understand the interrelationship between the sequence organization and thermodynamic property of the genomic sequence in the genomes of the four selected bacteria. Sequence data and the corresponding annotations were taken from GenBank genomes (ftp://ftp.ncbi.nlm.nih.gov/genomes/genbank/bacteria/). Shannon’s block entropy is used here to measure the digital information, whereas Gibbs’ entropy is employed to measure the analog information. Boltzmann probability distribution is used to convert the DNA stacking energies into probabilities for Gibbs entropy computations. To further relate the two forms of information to gene function, we also incorporated in our analyses the spatial distributions of the anabolic and catabolic classes genes. By doing so, we hoped to reveal the connections between analog and digital information types, as well as its possible functional meaning.

## Methods

First, in our study, the genome sequence is rearranged to start at the origin (OriC) of replication. Then, the entropy of chunks of the DNA sequence is computed by scanning the complete genome with a sliding window. To examine the effect of the window size, results are shown for window sizes of 100, 250, and 500 kb. Within a window, all possible words of the given block size (*N*) are counted. To account for all adjacent base interactions, neighboring base pairs are considered. That is, if the nucleotide sequence is “AGCTAG” and the block size is 3 base pairs (bp), AGC, GCT, CTA, and TAG are counted. In this section, the methodology is presented for a block size of three (*N*=3), other block sizes are handled likewise. The Shannon entropy quantifies the average information content of the sequence from the distribution of symbols (words) of the source [25]. It is mathematically given as

(1) $$ H_{N}=-\sum_{i}P_{s}^{(N)}(i) \log P_{s}^{(N)}(i)\;,   $$

where $P_{s}^{(N)}(i)$ is the probability (relative frequency) to observe the *i*th word of the block size *N* inside the window and the summation is over all possible nucleotide words of length *N*. Essentially, if we take a block size of 3 bp (i.e., codons), the sum will range up to 64. We count the frequency of every codon in the window and normalize it to the total number of codons. The Shannon entropy is maximal when all words occur at equal probabilities, and it is zero when one of the symbols occurs with probability one.

Boltzmann’s statistical explanation of the physical (thermodynamic) entropy relates it to the number of possible arrangements of molecules (microstates) belonging to a macrostate [26].

(2) $$ S_{\mathrm{B}}=k_{\mathrm{B}}\ln\Omega\;.  $$

*k*_B_ is the Boltzmann constant which gives this entropy a thermodynamic unit of measure, *k*_B_=1.38×10^−23^ *J*/*K*, and *Ω* is the number of accessible microstates. Boltzmann’s entropy is defined for a system based on a microcanonical ensemble in which the macrostate is of a fixed number of particles, volume, and energy. All states are accessed equally likely with the same energy [27]. Gibbs devised a generic entropy definition over the more general probability distribution of the possible states (canonical ensemble). The Gibbs entropy is defined as

(3) $$ S_{\mathrm{G}}=-k_{\mathrm{B}}\sum_{i}P_{\mathrm{G}}(i) \ln P_{\mathrm{G}}(i)\;,  $$

where the sum is over all microstates and *P*_G_(*i*) is the probability that the molecule is in the *i*th state. It can easily be seen that for a uniform distribution of states, the Gibbs entropy reduces to the Boltzmann entropy.

Gibbs’ entropy has a similar form as Shannon’s entropy except for the Boltzmann constant. Nevertheless, unlike the Shannon case where the probability $P_{s}^{(N)}$ is defined according to the frequency of occurrence, we associated the probability distribution with thermodynamic stability quantified by the nearest-neighbor free energy parameters. We used Sanatluca’s unified free energy parameters for di-nucleotide steps at 37 °C as in [23], presented here in Table 1. For block sizes greater than two, the energies are computed by adding the involved di-nucleotides. For instance, if the block size is three and the sequence is AGC, the energies of AG and GC will be added. This way, we have a list of codons with their corresponding energies, providing 64 energy states denoted by *E*(*i*). Assuming a random process behind the construction of the DNA, with a certain probability, one would obtain molecules with certain energies. If there are *n*_*i*_ codons in the *i*th energy state, we assumed that the probability for having a certain energy state follows the Boltzmann distribution given by

(4) $$ P_{\mathrm{G}}(i)=\frac{n_{i}e^{-\frac{E(i)}{k_{\mathrm{B}}T}}}{\sum\limits_{j}{n_{j}e^{-\frac{E(j)}{k_{\mathrm{B}}T}}}}\;.   $$

**Table 1:** Unified nearest-neighbor free energy parameters

Sequence	*Δ* *G*(Kcal/mol)
AA	−1.00
TT	−1.00
AT	−0.88
TA	−0.58
CA	−1.45
TG	−1.45
GT	−1.44
AC	−1.44
CT	−1.28
AG	−1.28
GA	−1.30
TC	−1.30
CG	−2.17
GC	−2.24
GG	−1.84
CC	−1.84

The thermodynamic stability parameters of Watson-Crick base pairs in 1 M NaCl at 37 °C [23]

*T* is the temperature in Kelvin. Although we are aware that the Boltzmann distribution gives the most probable distribution of energy (the one pertaining to the equilibrium state) for states having a random distribution of energies (e.g., ideal gas), which is not the case here, we just used it to have a representation of stability (energy) in an entropy-like expression.

**Fig. 1:**
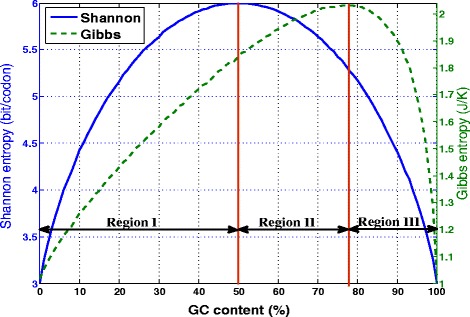
Shannon and Gibbs entropies as a function of GC content

To see how the Gibbs entropy captures the stability, we generated a random nucleotide sequence of length 100 kb with a specific GC content. By changing the GC content from 0 to 100 *%*, the Shannon and Gibbs entropies are calculated from the frequency distribution of the codons in the generated sequence. The result is shown in Fig. 1. The Shannon entropy function is symmetric with the maximum at 50 *%*. It tells us how random the sequence is. By comparing it with the maximum value, we can tell how diverse the sequence is, but it does not distinguish between AT and GC. However, except for larger GC content values (in region III), the Gibbs entropy curve is uniformly related to the GC content. If we are operating in regions I and II (the GC content of organisms typically cannot be greater than 80 *%*), the higher the Gibbs entropy, the higher the GC content, and hence, it measures stability. One has to be careful about the maximum point of the Gibbs entropy. The indicated maximum point in Fig. 1 is only valid for this randomly generated sample. For other realistic genome sequences, the maximum might move to elsewhere.

The functional gene groups were taken from the Gene Ontology (GO) tree provided by the RegulonDB database. Anabolic genes: biosynthesis of macromolecules (GID000000120); catabolic genes: degradation of macromolecules (GID000000057). To have a possibility of comparison between the bacteria, the orthologues of anabolic and catabolic genes were considered. The corresponding functional groups where counted in 500-kb sliding windows using a 4-kb shift. The window size was chosen so as to have a significant number of genes and obtain a smooth curve.

To support our qualitative statements of comparisons, localized Pearson correlation coefficients are incorporated in the figures. The local cross-correlation coefficients are calculated by taking 100 points to the left and right of the corresponding position. Pearson’s correlation coefficient between two vectors *x* and *y* is calculated as

(5) $$ r_{xy}=\frac{\sum\limits_{i=1}^{n}(x_{i}-\bar{x})(y_{i}-\bar{y})}{\sqrt{\sum\limits_{i=1}^{n}(x_{i}-\bar{x})^{2}} \sqrt{\sum\limits_{i=1}^{n}(y_{i}-\bar{y})^{2}}}\;,  $$

where $\bar {x}$ and $\bar {y}$ are the sample means of *x* and *y*, respectively.

## Results and discussion

### Shannon vs. Gibbs entropy on complete genomes

Our first aim was to compare the analog information, quantifying relative stability and measured with the Gibbs entropy (applying Boltzmann statistics to convert the stacking or melting energies to probabilities), with the digital Shannon information. To do so, the block size is set to 3 bp and a sliding window is shifted 4 kb at a time along the complete genome starting from the OriC as the center of the first window. The Shannon entropy is calculated using overlapping codons (i.e., with a shift of 1 bp).

**Fig. 2:**
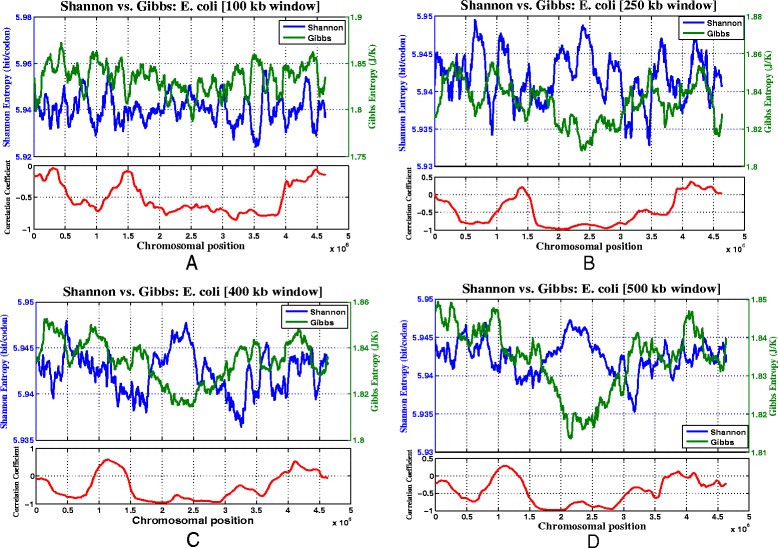
Shannon and Gibbs entropy profiles of *E. coli* for variable block sizes. Overlapping base pairs are considered. Block sizes are 2, 3, 4, and 5 bp in **a**, **b**, **c**, and **d**, respectively. The sliding windows (250 kb) are shifted 4 kb along the complete genome. The start and end positions are the origin of replication (OriC) whereas the terminus (Ter) is in the middle

**Fig. 3:**
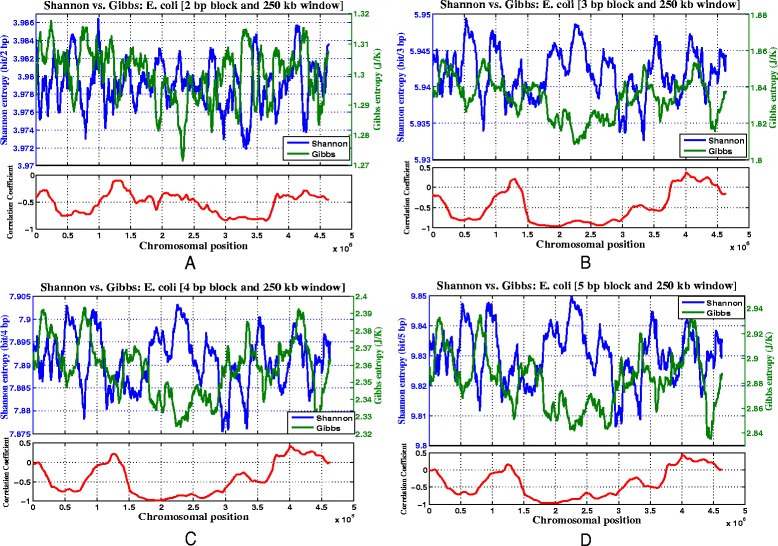
Shannon and Gibbs entropy profiles of *E. coli* for variable block sizes. Overlapping base pairs are considered. Block sizes are 2, 3, 4, and 5 bp in **a**, **b**, **c** and **d**, respectively. The sliding windows (250 kb) are shifted 4 kb along the complete genome. The start and end positions are the origin of replication (OriC) whereas the terminus (Ter) is in the middle

The Shannon and Gibbs entropies in the *E. coli* genome are plotted together for window sizes of 100, 250, 400 and 500 kb in Fig. 2. Since the nucleotide sequence is rearranged to start at the origin, the terminus region (Ter) will be exactly in the middle. This is also evidently visible from the shape of Gibbs entropy curve in which the lowest point is around the terminus, attributed to the AT-richness. Smaller windows lead to high fluctuations and are not easy to compare. Likewise, a very large window will hinder the visibility of the differences as a result of the smoothing effect it creates. Results for block sizes 2, 3, 4 and 5 bp and a window size of 250 kb are shown in Fig. 3. For 2 bp, the entropies are anti-correlated in all regions. The change to 3 bp has caused three regions to have a positive correlation, and these regions remain correlated in this way for higher block sizes. In addition, the vicinity of the terminus region has shown an extremely high anti-correlation. Similarly, there is no significant change when moving to 4 or 5-bp blocks. It is very significant that the overall shape of the curves as well as the positions of the troughs and crests remained unaffected by changes in both the block and window sizes.

The changes in entropy along the genome might seem very small. For example, in Fig. 3 b, the Shannon entropy in the *E. coli* genome (250-kb window) ranges from 5.9327 and 5.9494, which is a change of only 0.0167. To assess how significant the observed changes (*Δ*SE_observed_) are compared with the changes in entropy (*Δ*SE) in a random sequences, we have calculated the *Z*-score. However, the random model has to be selected in such a way that it preserves the biological sequence complexity as much as possible. Otherwise, any order present in the real genome will be lost and the resulting Shannon entropies will just be the maximum. We have fragmented the genome into genes and intergenic regions and produced 1000 random “genomes” by shuffling the positions of the fragments. For each random genome, the *Δ*SE is calculated and the distribution of shuffled *Δ*SEs is obtained. Finally, the *Z*-score of *Δ*SE_observed_ is obtained as (*Δ*SE_observed_−mean(*Δ*SE)/Std(*Δ*SE)). For *Δ*SE_observed_=0.0167 (Fig. 3 b), the *Z*-score is **3.74** and none of the randomized genomes have exceeded the *Δ*SE_observed_. This shows that the observed changes in entropies, even though very small, are highly significant and can safely be used to show differences in certain parts of the genome.

The two entropies are mostly anti-correlated in *E. coli*, with a stronger magnitude around the terminus. The terminus region is characterized by high Shannon entropy and low Gibbs entropy, that is, the sequence is more random and less stable. This means that the codon composition of the sequence has become slightly more balanced, which is due to an increase in AT-rich codons. Similarly, there are also positions where the Shannon entropy is relatively low and the Gibbs entropy is higher (e.g., in Fig. 2 around position 0.8 Mbp) which means a codon bias towards being more GC-rich. In general, our interpretation for a block size of 3 bp is that whenever both entropies increase, this means that both the GC content and the randomness have increased, and the sequence is more stable due to the usage of more GC-rich codons. However, if there is a decrease in the Gibbs entropy while the Shannon entropy is higher, the sequence has become less stable (AT-rich) and more random as a result of an increase in usage of AT-rich codons.

**Fig. 4:**
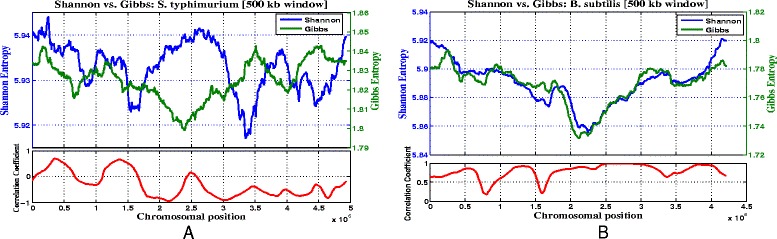
Shannon and Gibbs entropy profiles of *S. typhimurium* (**a**) and *B. subtilis* (**b**). The window size is 500- with 4-kb slide in both plots

The Shannon and Gibbs entropy profiles for *B. subtilis* and *S. typhimurium* for a window size of 500 kb are shown in Fig. 4. Since *S. typhimurium* and *E. coli* are close relatives in phylogeny, the Gibbs and Shannon entropy profiles in *S. typhimurium* show a behavior very similar to that in *E. coli* being mostly anti-correlated. In contrast, in the evolutionarily more distant gram-positive bacterium *B. subtilis,* the two entropies are highly correlated.

**Fig. 5:**
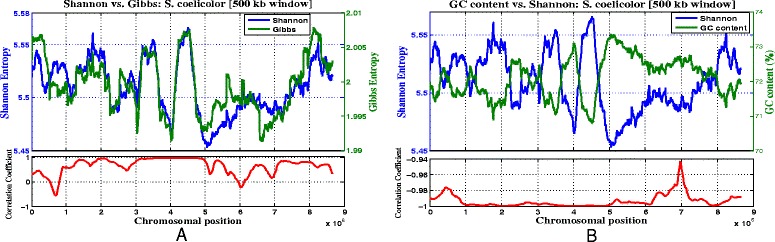
Shannon entropy of *S. coelicolor* with Gibbs entropy (**a**) and GC profile (**b**). The linear genome is rearranged in the oriC-Ter-oriC orientation

The relationship between the two entropies mostly depends on the GC content of the organism. This can be seen from Fig. 1. If the GC content is less than 50 %, there will be a direct relationship between the two. An increase in the number of AT-rich codons will reduce the Gibbs entropy (stability) and at the same time the Shannon entropy will decrease because of the skewed codon distribution. If Shannon entropy increases as a result of having more GC-rich codons, the Gibbs entropy will also increase. For organisms having a slightly more than 50 % average GC content, the entropies will have opposite behaviors. Most of the sequence will be slightly GC-rich and a further increase in GC content would mean an increase of Gibbs entropy. At the same time, the Shannon entropy will decrease as a result of the decrease in the variability of the sequence. *E. coli* and *S. typhimurium* have an average GC content of 51 and 53 %, respectively. Hence, for most regions, anti-correlation is observed. However, since the GC contents are in the vicinity of 50 % and locally it can be less than 50 %, the entropies may become positively correlated in some regions. For *B. subtilis*, however, the average GC content is 43.5 % and as a result the two entropies are entirely correlated with a global correlation coefficient of 0.9. In the region from the maximum point of Gibbs entropy to 100 % GC (region III in Fig. 1), as the stability (GC content) increases, the Gibbs entropy decreases. Therefore, the Gibbs entropy will not be in the same direction as the thermodynamic stability. The plot in Fig. 1 is done considering the codon distribution of a randomly generated sample sequence. However, for sequences containing mixtures of AT and GC, the maximum can be anywhere on the right-hand side. Therefore, when applying the Gibbs entropy measure on highly GC-rich genomes, one can end up in the last operating region where the Shannon and Gibbs entropies follow the same directions. This effect can be seen in Fig. 5 where the Shannon entropy profile of *S. coelicolor*, a highly GC-rich linear genome (average GC content is 72.12 %), is plotted with both the Gibbs entropy and the local GC profiles. The origin is located in the middle of the linear *S. coelicolor* but to be consistent with the plots of the other bacteria, the data is rearranged to have an orientation of OriC-Ter-OriC, although the actual genome is not arranged as a ring. The increase in the GC content makes the sequence more stable. Accordingly, both the Shannon and Gibbs entropies will decrease. Hence, one should mirror the Gibbs entropy to use it as a stability measure. The Shannon entropy is perfectly anti-correlated with the GC content and therefore the stability (see Fig. 5 b).

The Gibbs entropy also shows the spatial DNA sequence organization in the bacterial genomes. There is a gradient from the origin of replication to the terminus on both replichores with the most stable DNA near the origin and the least stable at the replication terminus. This pattern is consistent with the conserved gradient of DNA melting energy along the Ori-Ter axis in both replichores with a high average melting energy in the Ori-proximal region and a low average melting energy in the Ter-proximal region observed in *γ*-Protebacterial genomes [3, 8]. A similar pattern was found for the distribution of DNA binding sites for DNA gyrase, an enzyme introducing negative supercoils into the DNA [28–30]. It is assumed that the high concentration of gyrase binding sites in the Ori-proximal region creates a gradient of average negative superhelicity from high around OriC to low around Ter in both replichores. Another highly conserved pattern in *α*- and *γ*-Proteobacterial genomes is the gene order along the Ori-Ter axis [3, 4, 30]. The anabolic genes that are highly expressed during exponential growth are located in the vicinity of the origin of replication, whereas catabolic genes are predominantly located close to the terminus. These gradients of analog and digital information (DNA physicochemical properties and gene functions, respectively) have been related to the Ori →Ter directionality of DNA replication [4, 8], suggesting that the spatial organization of genomic DNA sequence is largely determined by the process of replication.

### Using Gibbs entropy for identification of coding and non-coding regions

**Fig. 6:**
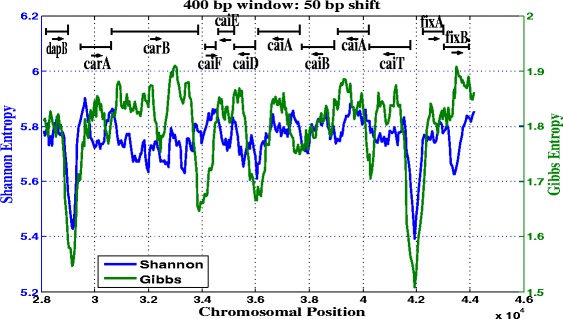
Shannon and Gibbs entropy profiles in a region of *E. coli* using a 400-bp window and a 50-bp shift. The 12 genes in the region are shown as lines at the top. Note that the troughs of the Gibbs entropy are exactly at the non-coding positions (i.e., gaps between genes)

The Gibbs entropy profiles can further be used as a tool for detecting non-coding and coding regions. Generally, because of the AT-richness of the promoters as well as the 5^′^ and 3^′^ gene flanking regions, the coding sequences are GC-rich compared to the corresponding non-coding sequences [8, 31]. Since the Boltzman probability distribution gives more weight to AT-rich sequences (see Eq. 4), the Gibbs entropy will have smaller values at the non-coding regions. We have used a smaller sliding window (400 bp) with a 50-bp shift on the region of the *E. coli* genome containing 12 genes. Figure 6 shows the result. The minimum low stability points clearly emphasize the non-coding sequence (the gaps between genes in the annotation at the top). Stability and melting temperature profiles have been previously used for identification of various genomic regions (e.g., see [32] and [33]). However, our method produces a significant variation in Gibbs entropy more clearly pointing out the differences in coding and non-coding regions of the genome.

### Shannon entropy in the protein coding sequences

So far, the Shannon entropy is computed considering overlapping triplets in the complete genomes. We now only take the protein coding sequences (CDS) of the four genomes and compute the Shannon entropy using both the distribution of the non-overlapping triplets (codons) and the corresponding translated amino acid distribution. In a given window, the protein coding genes in both strands are collected and the frequencies of the codons are counted. The base sequences of genes in the complementary strand are complemented and reversed before the counting so that the computed Shannon entropies reflect the actual codon and amino acid composition encoded in the region.

**Fig. 7:**
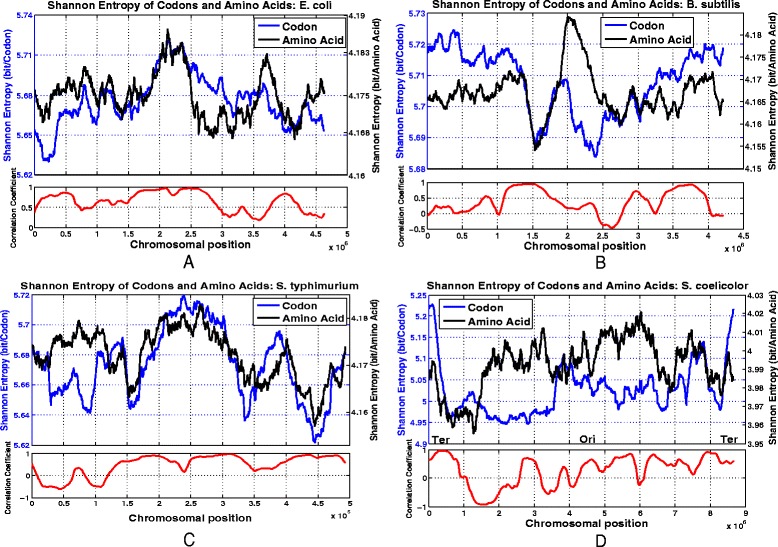
Shannon entropy profiles per codon and amino acid in the coding sequences of the four bacteria. *E. coli* (**a**). *B. subtilis* (**b**). *S. typhimurium* (**c**). and *S. coelicolor* (**d**). Window size is 500 kb

The codon-to-amino-acid translation is carried out using the standard genetic code. The results are shown in Fig. 7. Almost for all bacteria, the entropy profiles per codon and per amino acid positively correlate. However, there are regions where the two are negatively correlated (e.g., Fig. 7 b, d around positions 2.4 and 6 Mbp, respectively). The positive correlation can trivially be explained as a direct linear mapping between codons and amino acids. There is a certain level of expected positive correlation between the two profiles. However, since the number of codons encoding a similar amino acid (synonymous codons) varies (ranging from 1 to 6), a change in the frequency distribution of codons may not necessarily affect the amino acid distribution. In *E. coli* and *S. typhimurium*, a high Shannon entropy in the Ter-proximal region reflects the relatively more random nature of the codon and amino acid composition. Except for *S. coelicolor*, the terminus region has the highest amino acid entropy which means that the amino acid distribution in Ter region is more balanced.

**Fig. 8:**
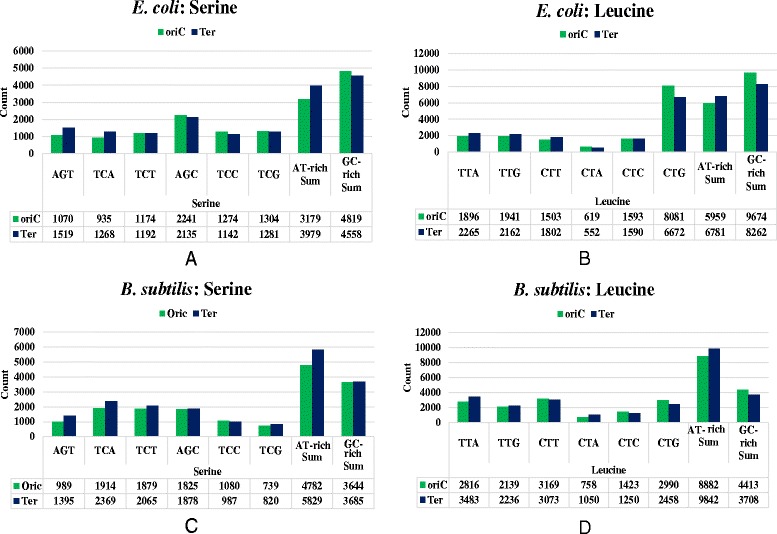
Synonymous codon usage in *E. coli* (**a** and **b**) and *B. subtilis* (**c** and **d**) at origin and terminus regions. AT-rich sum and GC-rich sum are the total number of AT and GC-rich codons, respectively

The regulatory sequence organization requirement of having an AT-rich terminus region and GC-rich origin is achieved by the selective usage of either synonymous codons or amino acids [9]. For example, the amino acid serine is encoded by AGT, TCA, TCT, AGC, TCC, and TCG. The first three codons are AT-rich whereas the last three are GC-rich. Similarly, the amino acids could also be classified as AT- and GC-rich. Amino acids such as proline, encoded by CCT, CCC, CCA, and CCG, can be regarded as a GC-rich amino acid. Likewise, lysine which is encoded by AAA and AAG could be regarded as an AT-rich amino acid. A less stable sequence around the terminus can be attained by using more AT-rich amino acids, which will in turn affect the distribution of amino acids (it will be biased towards the AT-rich ones) or the AT-rich codons among the synonymous ones without affecting the amino acid composition. In *E. coli* and *S. typhimurium*, the high Shannon entropy of codons and amino acids at the terminus (Fig. 7) indicates the more uniform codon as well as amino acid distributions. Thus, it appears that the less stable nature of the DNA in this region can be tolerated by allowing the synonymous codon usage. To reveal this selective codon usage, we counted the frequencies of the synonymous codons within two 500-kb windows, one located at the origin and another at the terminus. Here, only the non-overlapping triplets (codons) in the coding sequence are considered. Figure 8 a, b shows the synonymous codon usage in *E. coli* for amino acids serine and leucine. Note that in the Ter region, the frequency of the AT-rich codons has increased whereas that of the GT-rich ones have decreased. Although leucine is most often encoded by CTG, since it is a GC-rich triplet, its frequency has decreased considerably. This observation is pertinent also to the other amino acids. The terminus region of *B. subtilis* is also less stable and has the highest Shannon entropy of amino acids. Although the Shannon entropy of codons in the Ter region is not higher than around the origin, the selective usage of codons still occurs. As shown in Fig. 8, compared to the origin of replication, the frequency of AT-rich codons have increased in the terminus region. It is noteworthy that the low GC content of the organism by itself favors the use of AT-rich codons. For encoding serine and leucine, *B. subtilis* uses almost twice as many AT-rich codons as GC-rich ones (see Fig. 8 c, d). This justifies the observed low Shannon entropy of codons at the terminus region shown in Fig. 7 b.

### Relation to functional classes of genes

We have shown that the sequence organization is mainly dependent on the physicochemical property requirements to serve certain functions. For example, the less stable and hence AT-rich terminus region is assumed to absorb the positive superhelicity generated by the convergence of the two replisomes during replication [4]. We have also analyzed the spatial sequence organization in relation to other functional requirements. We chose two functional classes of genes—anabolic and catabolic genes—connected to energy and resource supply of the cell. Anabolic enzymes need energy to synthesize macromolecules. In contrast, catabolic enzymes degrade complex molecules in stages of energy and resource shortage.

**Fig. 9:**
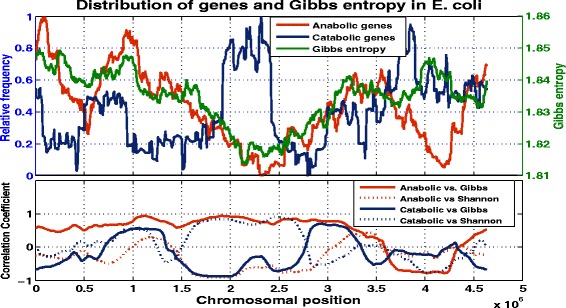
Distribution of anabolic and catabolic genes in *E. coli*. The correlations with Shannon and Gibbs entropies are also shown (500-kb window). The number of the genes relative to the total number of genes within the window is counted and normalized to [0;1] for equal scale visual display

The distribution of anabolic and catabolic genes of *E. coli* are plotted along with Gibbs entropy (thermodynamic stability) in Fig. 9. We used a 500 kb window and counted the number of genes of the corresponding functional group and normalized it to the total number of genes in the window. The gene frequencies are further normalized to 0 and 1 to plot them on a similar scale. Interestingly, the distribution of anabolic and catabolic genes are strongly related to the Gibbs entropy. Anabolic genes and Gibbs entropy are highly correlated (note the similarity in the profiles and also the magnitude of the correlation coefficient in Fig. 9). It seems that anabolic genes prefer sequences of high thermodynamic stability whereas catabolic genes prefer DNA encoding with low thermodynamic stability. Thermodynamically stable DNA sequences can only be used efficiently with the help of an extra energy input (e.g., to open up the DNA strands for transcription) and indeed, the anabolic genes are activated during the fast growth in rich medium. In this way, energy availability and an energy consuming functions are coupled [3]. In addition, anabolic and catabolic genes show an opposite chromosomal distribution pattern reflecting their antagonistic role in bacterial metabolism. There are two symmetric regions flanking the origin of replication (0.55 and 4.2) that show a deviation from the general pattern of a decreasing trend of anabolic genes concentration towards the terminus. These regions are known to harbor highly transcribed stable RNA (ribosomal RNA) genes. The transcription dynamics of stable RNA operons form large DNA structures called transcription foci [34], which may interfere with optimal thermodynamic coding for anabolic genes. The region at 4.1 Mbp appears even relatively enriched for catabolic genes, although the mutually exclusive genomic distribution of the anabolic and catabolic functions also holds in this region.

**Fig. 10:**
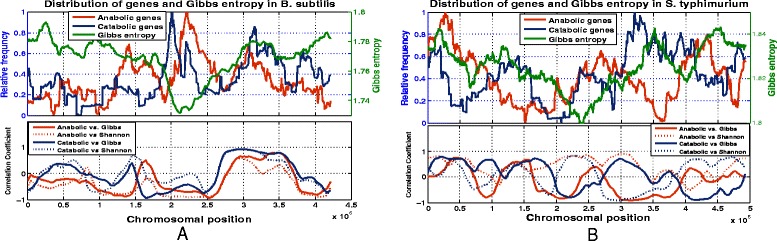
Distribution of anabolic and catabolic genes in *B. subtilis* (**a**) and *S. typhimurium* (**b**). The correlations with Shannon and Gibbs entropies are also shown (500 kb window). The number of the genes relative to the total number of genes within the window is counted and normalized to [0;1] for equal scale visual display

Similarly, we have looked into the distribution of the orthologues of anabolic and catabolic genes in *B. subtilis* and *S. typhimurium*. The results are presented in Fig. 10. In *B. subtilis*, at the terminus region, both anabolic and catabolic genes anti-correlate with the Gibbs as well as the Shannon entropies. The right replichore shows a very high correlation between the entropies and the functional classes of genes. At the terminus, although the sequence is less stable, a high number of both functional groups are observed, which is at variance with the results obtained in *E. coli*. However, since *B. subtilis* and *E. coli* have different lifestyles (e.g., occurrence of the process of septation in the former) and diverged about one billion years ago, substantial differences in genome organization are to be expected. The high correlation of Gibbs entropy and anabolic genes in *E. coli* supports the view that the genomic sequence organization is largely determined by the process of replication [4]. However, *B. subtilis* is known for its property of sporulation, which imposes constraints on the organization of the genome and chromosome segregation [35]. Also, it uses different replication factories and possesses different and much more numerous sigma factors [36]. Thus, we assume that the observed anti-correlation (Fig. 10 a) is due, at least in part, to these differences. The profiles of anabolic and catabolic genes of *S. typhimurium*, shown in Fig. 10 b, are also mostly anti-correlated with the Gibbs entropy. However, around the terminus region, catabolic genes are anti-correlated with the Gibbs entropy in all of the analyzed bacteria and although there is no ubiquitous relationship that explains how the functional groups are spatially organized, the obtained plots yield qualitative relations between digital and analog properties of the DNA sequence at specific sites in the chromosome.

## Conclusions

In addition to the digital type of linear genetic code encoding the proteins, DNA contains a continuous analog type of information resulting from the physicochemical properties of the DNA polymer. The analog information depends on the additive interactions of consecutive base steps rather than the individual bases. Hence, integrated analysis of the analog and digital DNA information types not only provides an additional angle to interpreting and understanding the genome sequence organization but also provides a way to integrate and consolidate the structural and functional data. In this study, we analyzed the relationships between the digital and analog properties of the DNA sequence with respect to the spatial organization of large functional classes (anabolic and catabolic) of genes in four bacterial species.

In *E. coli*, Shannon and Gibbs entropies are mostly anti-correlated. Especially, the two entropies are almost exactly opposite around the terminus. The results show that the global patterns of the entropies are more or less preserved independent of changing the window and block sizes. The observed gradient of Gibbs entropy from the origin to the terminus in both replichores is partly due to the GC content based selective usage of synonymous codons. The gradient of thermodynamic stability has been previously related to the process of replication and the demand to utilize the anabolic and catabolic genes at different stages of the growth cycle, facilitated by their location on the opposite chromosomal ends [1, 3, 4, 9]. Another core finding is the relation between the genomic distribution of anabolic and catabolic genes and the Gibbs entropy. In *E. coli*, anabolic genes are highly correlated with the Gibbs entropy whereas around the terminus region, catabolic genes are anti-correlated with Gibbs entropy in all analyzed bacteria. The observed patterns are very similar, implying a clear connection between functional gene types and DNA thermodynamic stability and, due to the correlation between entropies, also to statistical properties, i.e., the information content. We have also demonstrated the application of Gibbs entropy for the distinction of coding and non-coding regions based on the differences in DNA thermodynamic stability. While we propose this here, we think that verification of this proposal merits a separate study. The gram-negative enterobacterium *S. typhimurium* is closely related to *E. coli,* and therefore, it shows profiles very similar to *E. coli*. However, the AT-rich genome of the gram-positive soil bacterium *B. subtilis* exhibits different properties of organization. In *B. subtilis*, the Shannon and Gibbs entropy profiles are highly correlated. The distributions of the orthologues of anabolic and catabolic genes are also anti-correlated with the Gibbs entropy. *S. coelicolor* is a gram-positive bacterium with a lifestyle resembling fungi and containing two large plasmids in addition to the linear genome. The peculiarity of *S. coelicolor* is that the distribution of different types of genes reveals a central core comprising half of the chromosome and containing all the essential genes, whereas genes encoding apparently non-essential functions lie in the arms [37]. Notably, this biphasic structure of the chromosome does not align with the position of OriC. These peculiarities may affect the relationship between the analog and digital DNA information in organizing the genetic function in the highly GC-rich genome of *S. coelicolor*. Nevertheless, we observed that also in *S. coelicolor,* the Shannon entropy is perfectly anti-correlated with the GC content (Fig. 5 b). Taken together, our data strongly support the notion that the organization of the genetic code in the genome is dictated by thermodynamic properties of the genomic sequence. Digital and analog DNA information types are tightly intertwined parameters, which on evolutionary timescale, can adopt different relationships depending on the type and lifestyle of a bacterium.
